# Fabricating Microstructures on Glass for Microfluidic Chips by Glass Molding Process

**DOI:** 10.3390/mi9060269

**Published:** 2018-05-29

**Authors:** Tao Wang, Jing Chen, Tianfeng Zhou, Lu Song

**Affiliations:** 1 National Key Laboratory of Science and Technology on Micro/Nano Fabrication, Peking University, Beijing 100871, China; t.wang2010@pku.edu.cn (T.W.); l.song@pku.edu.cn (L.S.); 2 Key Laboratory of Fundamental Science for Advanced Machining, Beijing Institute of Technology, Beijing 100081, China

**Keywords:** glass molding process, groove, roughness, filling ratio

## Abstract

Compared with polymer-based biochips, such as polydimethylsiloxane (PDMS), glass based chips have drawn much attention due to their high transparency, chemical stability, and good biocompatibility. This paper investigated the glass molding process (GMP) for fabricating microstructures of microfluidic chips. The glass material was D-ZK3. Firstly, a mold with protrusion microstructure was prepared and used to fabricate grooves to evaluate the GMP performance in terms of roughness and height. Next, the molds for fabricating three typical microfluidic chips, for example, diffusion mixer chip, flow focusing chip, and cell counting chip, were prepared and used to mold microfluidic chips. The analysis of mold wear was then conducted by the comparison of mold morphology, before and after the GMP, which indicated that the mold was suitable for GMP. Finally, in order to verify the performance of the molded chips by the GMP, a mixed microfluidic chip was chosen to conduct an actual liquid filling experiment. The study indicated that the fabricating microstructure of glass microfluidic chip could be finished in 12 min with good surface quality, thus, providing a promising method for achieving mass production of glass microfluidic chips in the future.

## 1. Introduction

Microfluidic devices have been drawing a great deal of attention among academic and engineering communities due to their potential to revolutionize analytical measurements in chemical and biomedical areas [1]. They are much smaller, lighter, and cheaper than traditional instruments, and can improve efficiency and reduce reagents’ consumption dramatically. Various microfluidic devices are fabricated for various applications, such as capillary electrophoresis [2,3], semen testing [4], electrochromatography [5], and DNA separation [6].

To date, many substrates have been reported to fabricate microfluidic chips, such as silicon [7], polydimethylsiloxane (PDMS) [8], polymethyl methacrylate (PMMA) [9], and glass [10]. Silicon has good chemical and thermal stability, and can obtain complicated 2D and 3D microstructure by photolithography and etching approaches, but the downsides of fragility, high-cost, opacity, poor electrical insulation, and complex surface chemical properties impede its application. Contrastingly, PDMS and PMMA are widely used, in both academic and industrial fields, for biochips due to their high efficiency and low cost of fabrication process. However, they are not appropriate for certain applications, such as operating hydrophobic molecules or when stable surface characteristics are required, due to the issues of dissolution and surface property control [11]. Among them, glass is proved to be a more suitable substrate for microfluidic chips, which can provide advantages over other materials, such as beneficial optical properties, good insulating properties, high resistance to mechanical stress, high surface stability, and high solvent compatibility of the glass [12].

**Figure 1:**
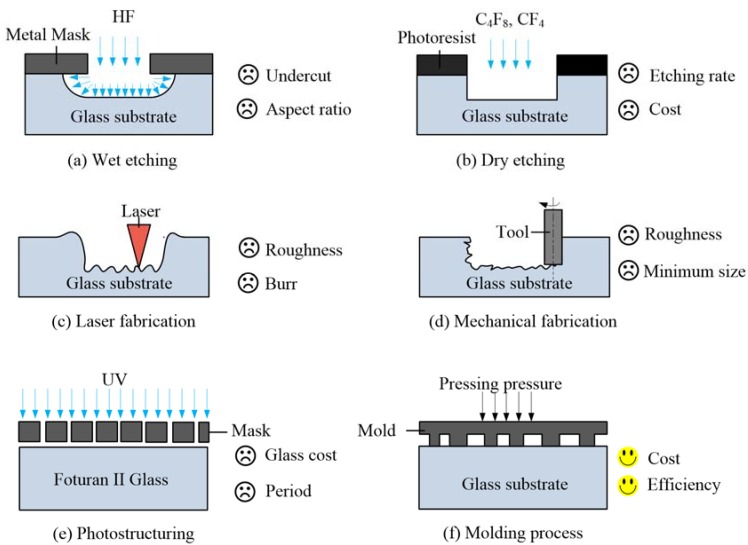
Six typical glass microstructure fabrication techniques. (**a**) Wet etching; (**b**) Dry etching; (**c**) Laser fabrication; (**d**) Mechanical fabrication; (**e**) Photostructuring; (**f**) Molding process.

However, fabricating the precise microstructure on glass for microfluidic chips is still challenging and many researchers have been exploring the field of glass microstructure fabrication techniques. Generally, glass microstructure fabrication techniques can be classified into six categories, as shown in Figure 1.

(1) Wet Etching

Glass is an isotropic material, which can be wet-etched by buffered hydrofluoric acid (HF) in a non-directional manner, as shown in Figure 1a. Wet-etching technique is well developed in fabricating microfluidic channels on glass [13,14,15,16] and it can achieve the production of microfluidic glass chips at a commercial scale at a reasonable cost. However, the wet-etching process suffers some inherent limitations, such as undercut, which exerts a challenge to fabricate high aspect ratio microstructure. In addition, the chemical liquid used in the process, especially buffered HF, is harmful to the environment, which needs further disposal before discharging.

(2) Dry Etching

Dry chemical etching of glass is carried out by capacitively coupled plasmas (CCP) [17], CCP/microwave plasma [18] and inductively coupled plasmas (ICP) [19]. It can achieve an anisotropic and precise profile, as shown in Figure 1b. Reactive ion etching results in an anisotropic profile due to the directional nature of the ion bombardment, affecting surface chemical reaction, as well as physical sputtering [20]. The technique for fabricating glass has been reported using different chemistries, such as C_3_F_8_, CHF_3_, CF_4_/CHF_3_, SF_6_/Ar and CF_4_/Ar [17,18,19,20,21]. The major weaknesses of glass dry etching are the low etch rate and the low etching selectivity of the glass to the etch mask, which impede its widespread application substantially.

(3) Laser Fabrication

Laser fabrication of microstructure on glass has been reported using CO_2_, UV, and ultra-short pulse lasers [22,23,24], and its mechanism is shown in Figure 1c. Although the efficiency of laser glass micromachining is much higher than those of other conventional methods, its wide application is hindered by the brittleness and poor thermal properties of glass, resulting in a risk of microcracking and poor surface quality on the bottom of groove [25].

(4) Mechanical Fabrication

The major mechanical fabrication techniques include micromilling [26,27], powder blasting [28,29,30], and micro-ultra-sonic machining [31]. They are superior in fabricating efficiency, despite it usually being a challenge to obtain smooth machined surfaces by mechanical fabrication. In addition, the minimum size of the microstructure mainly depends on the tool. 

(5) Photostructuring

Some photosensitive glass is commercially available, with the material itself being sensitive to ultraviolet (UV) light of a wavelength of around 310 nm (e.g., Schott Fortran [32]), which is amenable to anisotropic photostructuring. Therefore, it does not require an intermediate photoresist layer for patterning, as shown in Figure 1e. Although the fabrication process is simpler than the conventional wet-etching process, the whole processing period is still long at over 20 h. This is due to the required heating and cooling processes. In addition, the cost of Schott Fortran glass is greater than for conventional glass.

(6) Molding Process

Glass is a strongly temperature-dependent material. It is hard and brittle at room temperature, while it becomes a viscoelastic body or viscous liquid at high temperate. To date, the GMP has been accepted as a promising technique to efficiently generate microstructure on glass [33,34,35], as shown in Figure 1f. The main challenges are the microstructure fabrication on mold and the optimization of parameters for GMP.

Although GMP has been investigated in fabricating microstructure on glass for decades, the majority of the published reports focus on optical components [33,34,35,36,37,38], in which the typical microstructures are arrays of microgrooves, micropyramids, microneedles, microlenses, and microprisms. However, in reference to fabricating microfluidic chips, the typical microstructure is a U or rectangle cross-section groove. Recently, some researchers have started to shift the focus to the fabrication of microfluidic chips. Chen et al. [39,40] used nickel alloy mold to conduct their GMP for fabricating microfluidic chips in a conventional furnace, instead of a large-volume vacuum hot press. Since there is no water cooling system, the entirety of the GMP lasted more than 15 h. Huang et al. [41] utilized silicon molds to conduct the GMP in a GMP-207-HV (Toshiba Machine Co., Ltd., Shizuoka-ken, Japan) optical glass mold press machine for fabricating microfluidic chips, and the time of the GMP could, therefore, be reduced to less than 15 min. Since silicon material is inherently brittle, it is easy for fractures to occur during the GMP.

This paper explored the microstructure fabrication method of GMP on glass for microfluidic chips, which could lay an experimental and theoretical foundation for achieving mass production of microfluidic glass chips in the future. Since the glass–glass bonding process is an important issue for glass microfluidic chips, it will be investigated thoroughly in the future. Therefore, the chip verification in this paper was only conducted on a tape-glass bonding chip for simplification. In the present research, groove molding experiments were conducted and the molded profiles were observed. Moreover, the influence of the molding parameters was investigated, with corresponding experiments investigating the molding of microfluidic chips also being conducted and the molded chips were demonstrated. Finally, issues surrounding the curved side wall and bonding technique were discussed.

## 2. Experiments

### 2.1. Experimental Setup and Measurement

**Figure 2:**
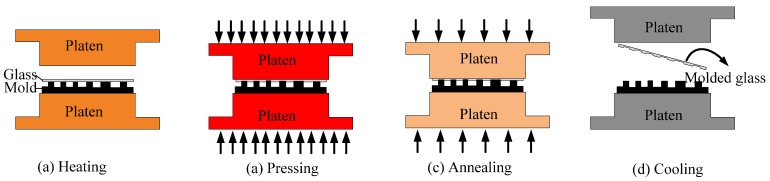
The schematic photo of the GMP. (**a**) Heating; (**b**) Pressing; (**c**) Annealing; (**d**) Cooling.

**Figure 3:**
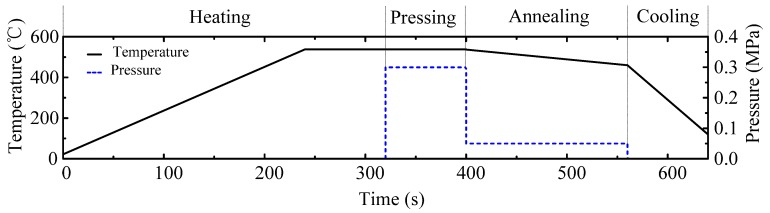
The typical evolution of temperature and pressure during the glass molding process (GMP).

All experiments were carried out on an ultraprecision glass molding machine, PFLF7-60A (SYS Corp., Tochigi, Japan), which could operate between 20–750 °C The glass molding process is schematically illustrated in Figure 2. The GMP consists of four steps: Heating, pressing, annealing, and cooling. In the first step, the glass and mold are placed on the bottom platen and heated together until the molding temperature is reached, which is typically 10 °C above the transition temperature of the glass material, as shown in Figure 2a. Next, the upper platen is driven downward to conduct the pressing process, which achieves the replication of the microstructure from the mold onto the preform. Then, the temperature is slowly cooled down to somewhat below the annealing point, while a small pressing pressure is used to maintain high fidelity, as shown in Figure 2c. Finally, the preform experiences the fast cooling step via the water cooling system and the microstructure is obtained by demolding, as shown in Figure 2d. The typical evolution of temperature and pressure during the GMP in experiment is shown in Figure 3.

The surface morphology of the mold was observed by a VK-X200 3D Laser Scanning Microscope (Keyence Corporation, Osaka, Japan) and FEI Quanta 250 FEG (Thermo Fisher Scientific, Waltham, MA, USA). The surface morphology of the molded glass was studied by an Olympus Lext OLS4100 (Olympus Corporation, Tokyo, Japan) and all the roughness measurements were conducted by this as well. 

### 2.2. Glass and Mold

The D-ZK3 optical glass wafer, with a 25.4 mm diameter and 0.6 mm thickness, was used in the experiment. The material is dense barium crown optical glass manufactured by CDGM GLASS Co., LTD (Chengdu, China). The major chemical compositions and thermal properties are listed in Table 1 and Table 2, respectively. *T*_g_ is defined as the transition temperature, above which the volume expanding rate increases abruptly as the temperature increases. *T*_s_ is defined as the yielding temperature, at which the glass reaches its maximum expansion point and starts shrinking as temperature increases further. In order to shorten the paper length, the morphology details of molds are listed in Figures S1–S5 in Supplementary Materials.

### 2.3. Design of Experiment

The main factors influencing the molded morphology are the pressing temperature, pressure, and time. The pressing temperature is usually set 10 °C above the transition temperature, *T*_g_, according to previous experiments. In this paper, the pressing temperature range was set between 547 °C and 552 °C, which means that the adhesion between the glass and the mold was avoided and demonstrates the influence of temperature. In regards to pressure, this was set between 0.1 MPa and 0.5 MPa, which is the main molding pressure in the molding machining. Pressing times was set as 80s in all experiments as its influence is insignificant if the time is above 60 s, based on our previous research. The present experiment consisted of two parts. The first part was the fundamental study investigating the influence of molding parameters, such as temperature and pressure, on the morphology of the molded groove, which can help to ascertain the optimal parameters. The second part was the investigation of the microstructure morphology during the fabricating of microfluidic chips by the GMP. The details of the parameters in the experiment design are listed in Table 3.

## 3. Results and Discussion

### 3.1. Groove Molding Experiment

**Figure 4:**
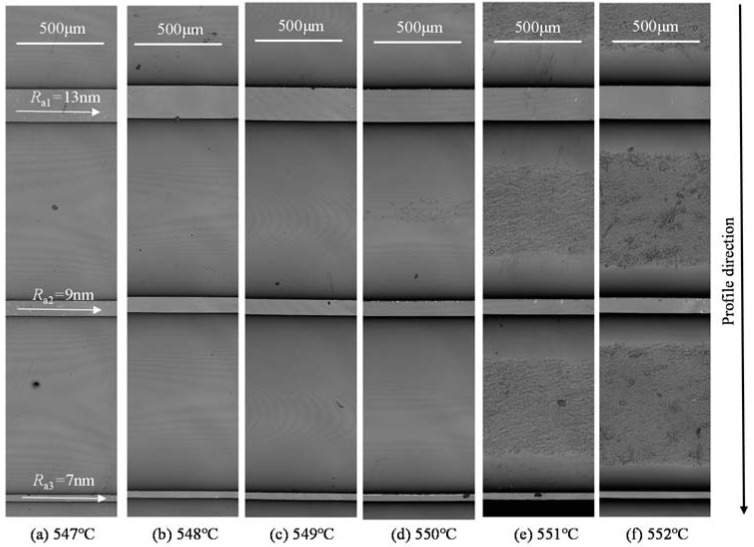
The molded grooves at different molding temperatures (*P* = 0.3 MPa). (**a**) 547 °C; (**b**) 548 °C; (**c**) 549 °C; (**d**) 550 °C; (**e**) 551 °C; (**f**) 552 °C.

The aim of this study was to investigate molding performance during the fabricating of grooves with a 60 μm depth. The molded grooves at different molding temperatures are shown in Figure 4. There are three grooves with a width of 200 μm, 100 μm, and 50 μm, respectively in each photo. All bottom surfaces of the grooves are extremely smooth. In order to get the quantitative value of the surface quality at the groove bottom, the sample molded at the temperature of 547 °C was taken and the surface roughness measurements were taken of all bottom surfaces running parallel to the grooves by the Olympus Lext OLS4100 (Olympus Corporation, Tokyo, Japan), as shown in Figure 4a. The results demonstrate that the surface roughness, *R*_a_, with the width of 200 μm, 100 μm, and 50 μm, are 13 nm, 9 nm, and 7 nm, respectively. Although the results somewhat indicate a correlation between the channel width and the mold surface roughness, its value is mainly determined by the mold surface roughness due to the contact between the mold surface and the groove bottom surface during the molding process. Due to the polishing process, the surface roughness, *R*_a_, of the mold can achieve around 10 nm. However, as an exploring investigation, the molding experiment was not conducted in a clean room and the individual roughness value may be influenced by some dust on the groove bottom surface, leading to small variations in the measurements at different locations.

It is worth mentioning that the bottom of the groove fabricated by the GMP is much better than those of the conventional fabricating methods, which is beneficial to the reduction of residual bio-samples and the increase of light transmittance performance. In addition, when temperature increases to 551 °C, some rough zones can be observed between grooves, and as temperature increases further, the size of the zones increase correspondingly. Since the surface roughness of the mold during the GMP is relatively rough (*R*_a_ around 0.3 μm) due to its fabricating process, it is expected that this zone touched the bottom of the mold during the GMP.

**Figure 5:**
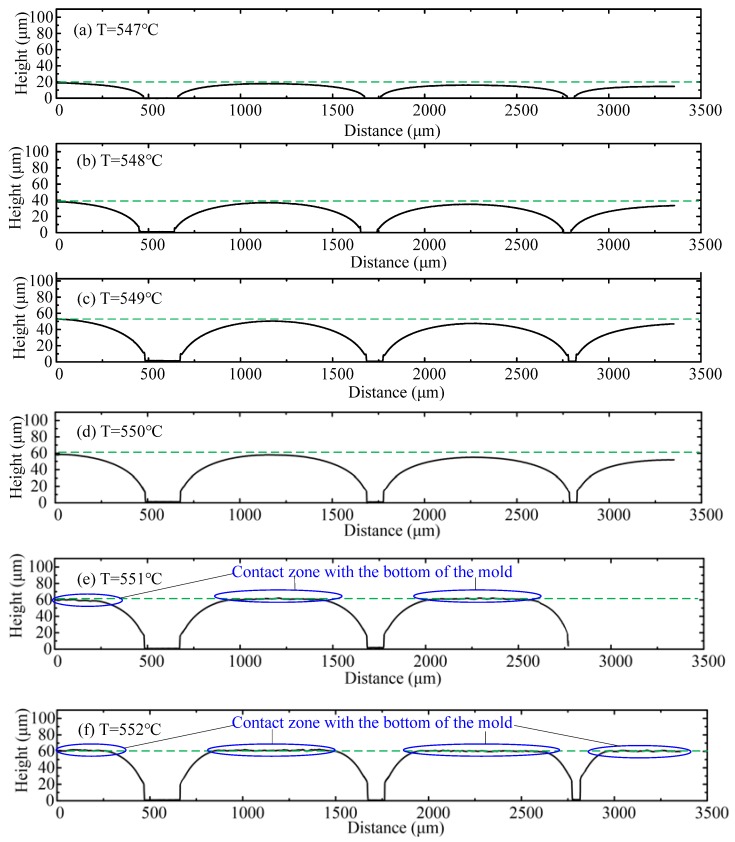
The profiles of the molded grooves at different molding temperatures. (**a**) 547 °C; (**b**) 548 °C; (**c**) 549 °C; (**d**) 550 °C; (**e**) 551 °C; (**f**) 552 °C.

In order to provide more information about the molded grooves, the profiles perpendicular to the grooves were extracted in each sample, as shown in Figure 5. It is evident that the depth of the groove increases with the molding temperature, from 40 μm at 547 °C to 60 μm at 550 °C. The corner radius of the grooves decreases with the molding temperature also. In addition, when the temperature reaches 550 °C, the glass almost touches the mold bottom and when it increases further, the contact zones in the profiles can be observed as the rough and horizontal areas on the top, as shown in Figure 5e,f.

**Figure 6:**
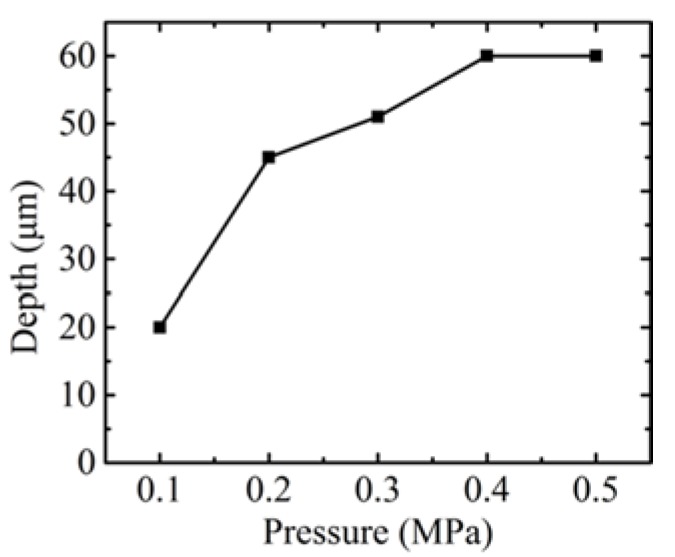
The influence of pressure on depth.

**Figure 7:**
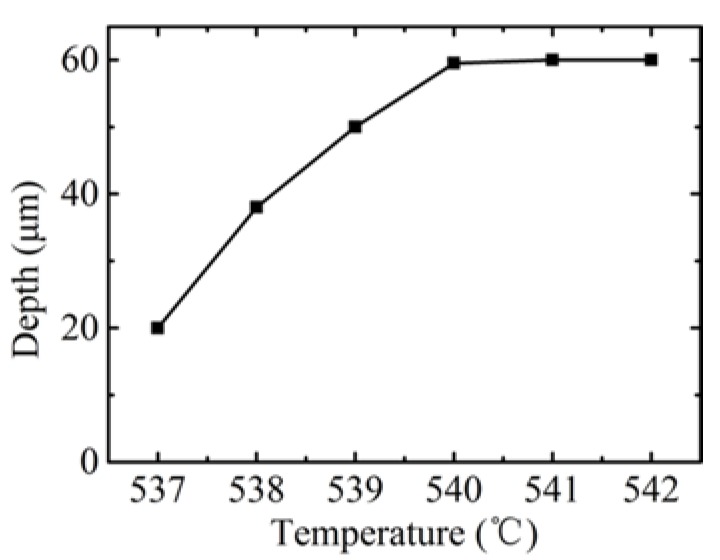
The influence of temperature on depth.

**Figure 8:**
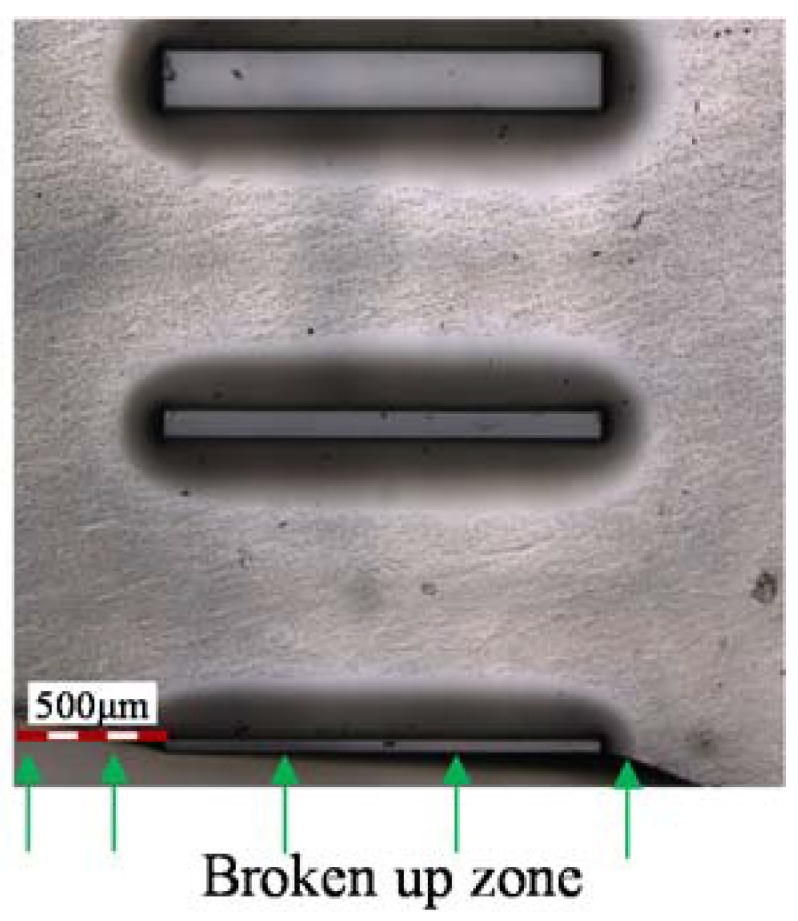
A broken glass.

The influence of the pressure is similar to that of the temperature. In order to avoid redundancy, the microstructure images and profiles molded at different pressures are ignored in the paper. The influence of the molding parameters, such as pressure and temperature, on groove depth are shown in Figure 6 and Figure 7, respectively. It indicates that the groove depth increased with both parameters. The depth value reached was maximum at a pressure of 0.4 MPa, with the temperature set at 549 °C, and at 550 °C when the pressure was set at 0.3 MPa. It is worth noting that, although further increases of both parameters would benefit the fill ratio, this increases the risk of cracking in the glass, in addition to adhesion between the mold and the glass. Therefore, the investigation of the optimal parameters for the maximum filling ratio are not included in the scope of the paper. Nevertheless, all the parameters of temperature and pressure used in this experiment were sufficient to avoid adhesion during the demolding process. The phenomenon of typical broken up in glass after demolding is shown in Figure 8. The whole part of the bottom material breaks away from the top material, which should be avoided in the GMP.

**Figure 9:**
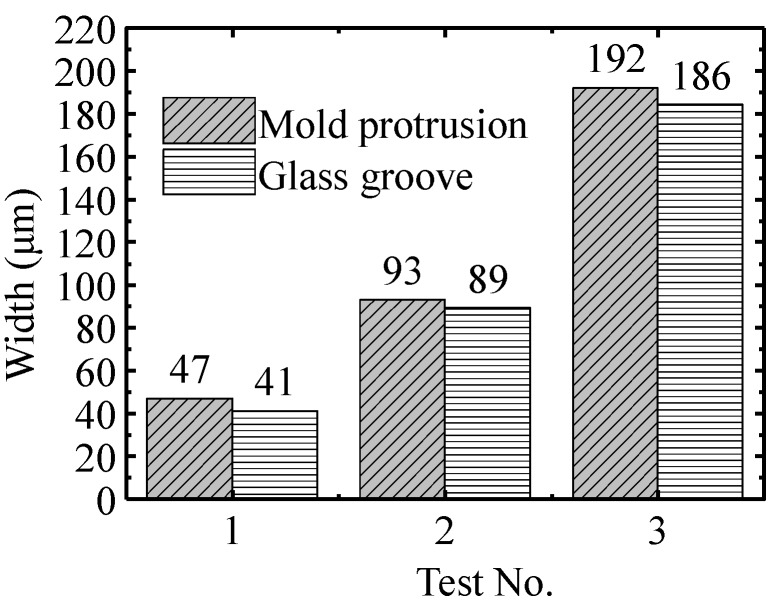
The width comparison between mold protrusions and glass grooves.

In order to reveal the accuracy of the molded glass grooves relative to the actual mold features, the width comparisons between mold protrusions and glass grooves are shown in Figure 9. The sample of glass groove data is extracted from the profile at a temperature of 552 °C, which is demonstrated at the bottom of Figure 5. It indicates that the widths of mold protrusions are all wider than the corresponding widths of glass grooves, and that the molding accuracy was around 4–6 μm. The different widths can be attributed to the fact that, when the force from the protrusions compressing the glass preform is taken off by demolding, the walls of the glass grooves experience springback, therefore, leading to the smaller glass groove width. 

### 3.2. Microfluidic Chips

**Figure 10:**
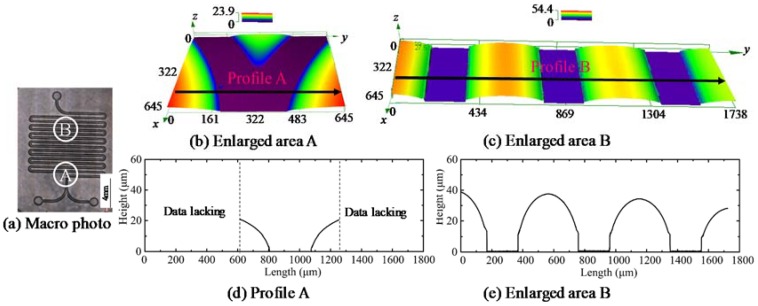
The microstructure of the molded diffusion mixer chip (550 °C, 0.5 MPa). (**a**) Marco photo; (**b**) Enlarged area A; (**c**) Enlarged area B; (**d**) Profile A; (**e**) Enlarged area B.

A typical microstructure of a molded diffusion mixer chip is shown in Figure 10. The macro photo indicates that the basic morphology of the diffusion mixer chip was achieved. In order to provide more details of the molded chip, area A (which is the combination area of the two input channels) and area B (which is in the typical U-shape mixed area) were enlarged, as is shown in Figure 10b,c, respectively. Since it is a small area, the highest depth was only 23.9 μm in Figure 10b. The Y shape was formed, which is suitable for the two liquids combining. In order to provide more details of the morphology, the profile extraction was conducted along the location, as shown in Figure 10b, and the profile is shown in Figure 10d. Although the corner radius is relatively large, it can be reduced by further parameter optimization. In addition, it is also valuable if there are some round corners in the cross-section profile of the channel in microfluidic chips. Regarding the mixing zone, this profile was also extracted, and is shown in Figure 10e. It indicates that the channels running parallel to each other, with a depth of 40μm, can be formed by the GMP, which is deep enough for the mixing function.

**Figure 11:**
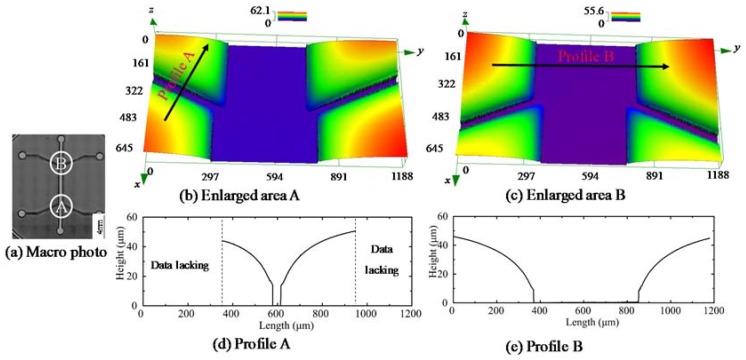
The microstructure of the molded flow focusing chip (550 °C, 0.5 MPa). (**a**) Macro photo; (**b**) Enlarged area A; (**c**) Enlarged area B; (**d**) Profile A; (**e**) Profile B.

A typical microstructure of the molded flow focusing chip is shown in Figure 11. The macro photo indicates that the basic morphology of the flow focusing chip was achieved, as shown in Figure 11a. In order to provide more details about the formed microstructure, the area A and area B were enlarged, as shown in Figure 11b,c, which are the combination of the three channels at the bottom and top, respectively. They both demonstrate that the expected channels’ morphology was generated, which was deep enough for the flow focusing experiment. The extracted profiles are shown in Figure 11d,e, and the section profiles are both characterized by a large corner radius.

**Figure 12:**
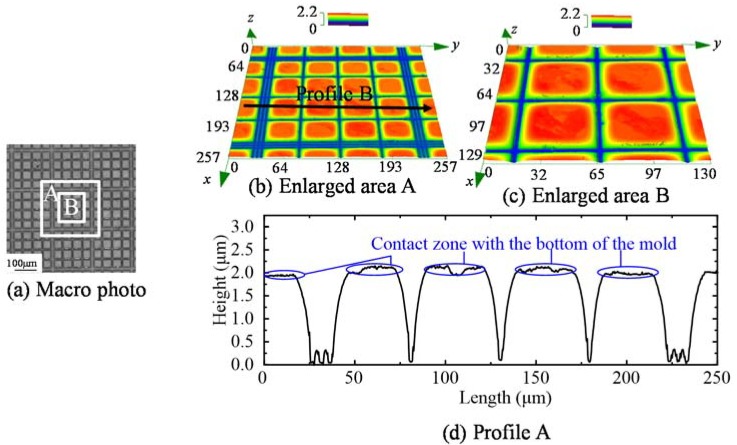
The microstructure of the molded cell counting chip (550 °C, 0.5 MPa). (**a**) Marco photo; (**b**) Enlarged area A; (**c**) Enlarged area B; (**d**) Profile A.

A typical microstructure of the molded cell counting chip is shown in Figure 12. The macro photo indicates that the basic morphology of the cell counting chip was achieved, as shown in Figure 12a. The enlarged images of area A and area B are shown in Figure 12b,c, respectively. The profile extracted from Figure 12b is shown in Figure 12d. Although the line width was only 1.5 μm on the mold, the molded groove is clear, with a depth of approximately 2 μm. Due to the contact between the glass and the mold bottom, the top of the chip was relatively rough, which is beneficial for cells to stay inside. According to currently morphology, the molded glass is potentially valuable for cell counting.

### 3.3. Chip Application

In order to verify the performance of the molded chips by the GMP, a mixed microfluidic chip was chosen to conduct the filling microfluidic system experiment. In terms of the bonding material, ARseal PSA clear polypropylene film tapes (MH-90697, Adhesives Research, Inc., Glen Rock, PA, USA) were used for their simplicity, and was coated with an inert silicone adhesive. The tapes provide an immediate bond to most materials and can also provide a barrier for preventing evaporation and cross contamination.

**Figure 13:**
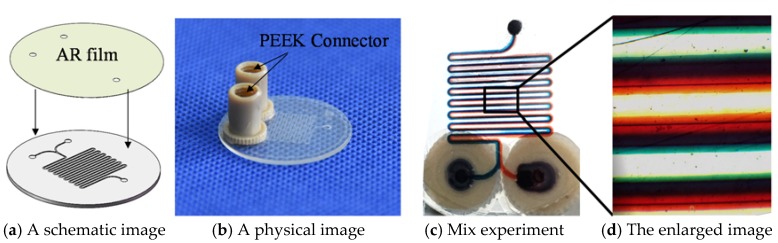
The experiment of filling the mixing microfluidic system. (**a**) A schematic image; (**b**) A physical image; (**c**) Mix experiment; (**d**) The enlarged image.

The experiment of the filling microfluidic system is shown in Figure 13. Firstly, the corresponding liquid inlet and the outlet hole were fabricated by a laser cutting machining and the bonding process was carried out in a clean room, as shown in Figure 13a. Next, the polyetheretherketone (PEEK) connectors were bonded with the chip by glue, which helped to locate the inlet holes during the filling with liquid, as shown in Figure 13b. Next, two inlet holes were filled with the red and blue ink by a pump at the same filling speed of 2 μL/min. The filling status is shown in Figure 13c and the enlarged image of the mixed channel zone is shown in Figure 13d. It was shown that the two reagents can flow along the channel without cross contamination occurring, and the obvious stratification phenomenon was observed. This proves that the molded chip can be used in a microfluidic system.

## 4. Discussions

### 4.1. Curved Side Wall

Currently, only the curved sidewall of the microchannel could be achieved by the GMP in this experiment. Since the cross-section of the microstructure on the mold is rectangle, it is possible to obtain a side wall of the channels on the molded glass that is close to 90 when appropriate parameters are set. However, this scenario did not occur due to the severe adhesion problem between the mold and the glass in the actual experiment. A promising direction is the utilization of the ultrasonic vibrations during the GMP, which is worth exploring in the future.

Although the curved side wall generated by the GMP is somewhat similar to the morphology obtained by the wet-etching, the GMP has obvious advantages over the wet-etching in several aspects, which are detailed below:

(1) Higher efficiency in the fabricating of a single microfluidic glass chip

The wet-etching process consists of cleaning, lithography, and buffered oxide etch (BOE) etching, which needs several instruments and is longer than 1 h [43]. This process can achieve the production of microfluidic glass chips at a commercial scale on a regular basis and at reasonable costs. However, for the fabricating of a single microfluidic glass chip, the time of the GMP is usually less than 15 min, which is much shorter than that of the hydrofluoric acid (HF) etching.

(2) More eco-friendly

The chemical liquid used in the wet-etching process, especially buffered HF, is harmful to environment, while only nitrogen is utilized in the GMP as a protecting gas, which is significantly more eco-friendly.

(3) Wider fabricating capacity

Some researchers [13,14,15,16] have found that it is typically hard for the wet-etching process to generate a microstructure with a depth-to-width ratio that is higher than 0.5, while the GMP has wider fabricating capacity than the wet-etching. For instance, a microstructure with a depth-to-width ratio of one has been achieved by the GMP [44].

In terms of the application of channels with a curved side wall, many researchers have conducted biochemical tests on these. Lin et al. [43] demonstrated a micro flow-through sampling glass chip, which was fabricated by wet-etching, and the side walls of the channels were curved. The chip sampled and separated Cy5-labelled BSA (Nanocs Inc., New York, NY, USA) and anti-BSA successfully. Lee et al. [45] demonstrated a microcapillary electrophoresis (μ-CE) device for DNA separation and detection, and the channels with curved side walls on the plastic chip were fabricated by the hot embossing method. Castaño-Álvarez et al. [46] fabricated glass microfluidic channels with curved side walls by photolithography and wet-etching. The microfluidic chips were successfully used, in combination with a metal-wire end-channel amperometric detector, for capillary electrophoresis (CE). Evander et al. [47] fabricated glass microchannels with curved side walls by wet-etching, and the glass microfluidic chip was used for acoustic force control of cells and particles in a continuous microfluidic process. Nevertheless, although the side walls of the channel fabricated by the GMP were curved, currently, the chips are still valuable for many applications.

### 4.2. Bonding Techniques

Generally, there are three categories of glass bonding techniques: (1) Direct bonding, involving surface-activated or fusion bonding; (2) Anodic bonding, with silicon as the intermediate layer; and (3) Adhesive bonding, with additional adhesive material. The strengths and weaknesses of each method can be found in [48]. In this paper, since the focus is on investigating the new material performance in the GMP, the bonding process was achieved by a simple method of the glass microchannel being sealed by a polymer adhesive sheet. Compared with the three traditional methods, the bonding process in this paper has the following two advantages:

(1) High efficiency

Compared with direct and anodic bonding, the polymer adhesive sheet bonding can be achieved in room temperature without any additional chemical solution and, subsequently, there is no clogging problem, which usually occurs in adhesive bonding. Thus, it is much faster to achieve the prototyping of the microfluidic chips for research.

(2) Recycling value

The bond of failed or used bonded microchips can easily be broken by soaking the microchips in acetone, with sonication, without damaging the glass microstructure, and they can be reused by the polymer adhesive sheet bonding process.

**Figure 14:**
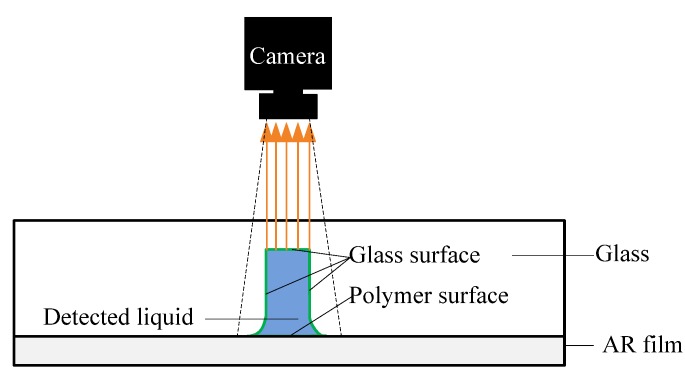
A typical testing diagram of the produced glass and anti-reflection (AR) film chip.

Although it is a simple method, it is worth pointing out that the bonding process in this paper is assumed to not compromise the main advantages of the glass microfluidic chips over polymer chips, such as biocompatibility, optical transparency, and surface modification. As for biocompatibility, since the anti-reflection (AR) film is designed to be biocompatible, the influence of the AR film is supposed to be small enough. In terms of optical transparency, it is true that the polymer AR film may exert some negative influence on this aspect. However, this issue can be resolved if the test is conducted by placing the chip upside down, as shown in Figure 14. When the surface modification is concerned, since there are still three glass surfaces left in the microchannel, different surface modification procedures can be conducted as well, such as protein immobilization [49]. Therefore, the microfluidic chip assembled by polymer adhesive sheet bonding is still valuable for practical applications.

## 5. Conclusions

This paper investigated the fabricating of glass microfluidic chips by the GMP, which provided a promising method for fast prototyping and mass production of microfluidic chips in the future. The main conclusions were as follows:

(1) A groove with a 60 μm height could be obtained by the GMP in 12 min, and the bottom roughness, *R*_a_, could be as small as 10 nm. The molded groove depth increased dramatically with the molding temperature and pressure before the time when the glass contacted with the bottom of the mold. Precautions should be taken to avoid cracks generated inside the glass.
(2) The microstructure of a diffusion mixer chip and a flow focusing chip were generated with more than 40 μm groove depths by the GMP, and the microstructure of the cell counting chip was fabricated with the groove of a 2 μm depth and 1.5 μm width.
(3) The study of mold wear indicated that the macro shape of the microstructure on the mold changed little over the time of 20 experiments, although the edge became a little blunt due to the weak stiffness in this area. The results from the energy-dispersive X-ray spectroscopy (EDS) analysis found that there was no chemical wear.
(4) The performance verification of the molded chips was conducted on a mixed microfluidic chip and the result indicated that the two reagents could flow along the channel without cross contamination, and that the obvious stratification phenomenon was observed. This proved that the molded chip could be used in a microfluidic system.

## Figures and Tables

**Table 1:** The major thermal properties of D-ZK3 glass [42].

Content	*T*_g_ (°C)	*T*_s_ (°C)	*T*_10_^14.5^ (°C)	*T*_10_^13^ (°C)	*T*_10_^7.6^ (°C)	α_100/300 °C_ (10^−7^/K)
Value	511	546	471	499	605	93

**Table 2:** The chemical compositions of D-ZK3 glass [42].

Content	SiO_2_	B_2_O_3_	CaO	Al_2_O_3_	BaO	Sb_2_O_3_
Value	30–40%	20–30%	0–10%	0–10%	10–20%	0–10%

**Table 3:** Details of the parameters in the glass molding process (GMP).

Item	No.	Temperature (°C)	Pressure (MPa)
Varying temperature	1	547	0.3
	2	548	0.3
	3	549	0.3
	4	550	0.3
	5	551	0.3
	6	552	0.3
Varying pressure	7	549	0.1
	8	549	0.2
	9	549	0.4
	10	549	0.5
Diffusion mixer chip	11	550	0.5
Flow focusing chip	12	550	0.5
Cell counting chip	13	550	0.5

## Supplementary Materials

The following are available online at http://www.mdpi.com/2072-666X/9/6/269/s1, Figure S1: The morphology of the mold for fabricating channels on glass, Figure S2: The morphology of three different molds for fabricating microfluidic chips, Figure S3: The mold morphology after GMP, Figure S4: The microstructure of molds before GMP, Figure S5: The microstructure of the molds after GMP.
