# Supplementary material for: Fabricating Microstructures on Glass for Microfluidic Chips by Glass Molding Process
Source: Micromachines (Basel). 2018 May 29;9(6):269. doi: 10.3390/mi9060269 (PMC6187291; doi:10.3390/mi9060269)
Supplement: Supplementary file 1 [file micromachines-09-00269-s001.pdf]

Supplementary Materials: Fabricating Microstructures on Glass for Microfluidic Chips by Glass Molding Process

Tao Wang, Jing Chen *, Tianfeng Zhou * and Lu Song

1. Microstructure of Molds before GMP

The molds fabricated by lithography and inductively coupled plasma deep reactive ion etching (ICP-DRIE) are provided by HICOMP MicroTech (Suzhou, China) Company Limited. Basically, the fabrication process of the mold microstructure consisted of two steps: firstly, the lithography was conducted to define the pattern; then the molds were etched directly. The minimum feature size could be obtained is 1μm. The roughness R_a of the top mold surface and the etched surface are around 10nm and 0.3μm, respectively. The microstructure could satisfy the general requirements of the mold for fabricating glass microfluidic chips.

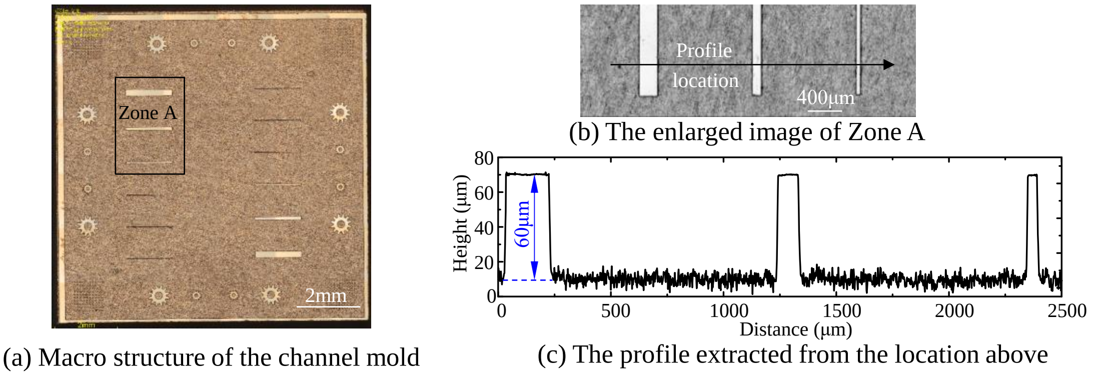

Figure S1. The morphology of the mold for fabricating channels on glass. (**a**) Macro structure of the channel mold; (**b**) The enlarged image of Zone A; (**c**) The profile extracted from the location above.

Figure S1 demonstrates the mold used for fabricating channels in the glass. The length, width and height of the mold are 10mm, 10mm and 2mm, respectively. The macro structure of the mold is shown in Figure S1(a). In this paper, the molded groove by only width of 192μm, 93μm and 47μm were studied due to their steady shapes, as shown in Zone A, and their design sizes were 200μm, 100μm and 50μm, respectively. In addition, their heights are 60μm which is assumed to be deep enough for general application in the microfluidic chips. The enlarged image and profile are shown in Figure S1(b) and Figure S1(c), respectively.

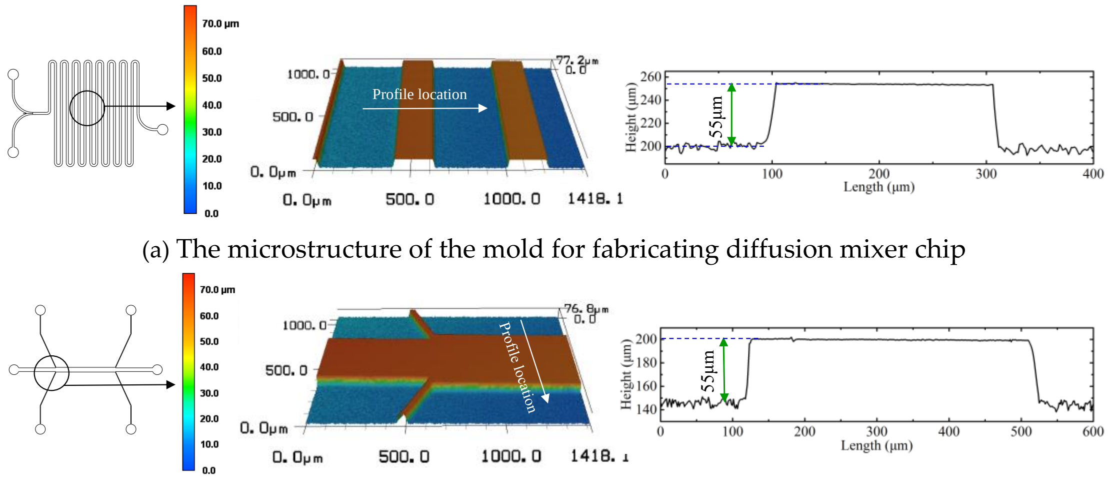

(b) Flow focusing chip

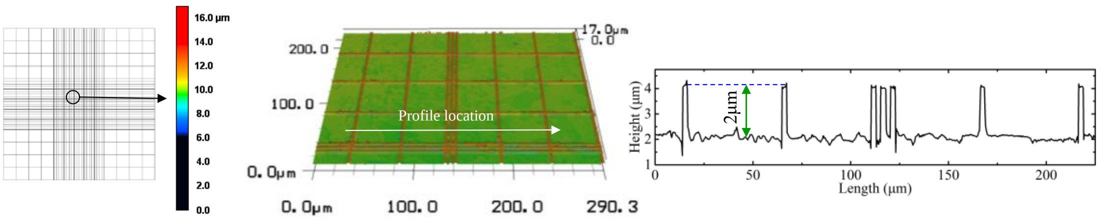

(c) Cell counting chip

Figure S2. The morphology of three different molds for fabricating microfluidic chips. (**a**) The microstructure of the mold for fabricating diffusion mixer chip, (**b**) Flow focusing chip and (**c**) Cell counting chip.

Due to the requirement of various types of bio-experiment, there are diverse functions which necessitate different design patterns. In this paper, fabricating three typical chips, i. e., diffusion mixer chip, flow focusing chip and cell counting chip, were studied, and the corresponding molds are shown in Figure S2(a), S2(b) and S2(c), respectively. Their macrostructures are demonstrated in the left, while the enlarged 3D images are located in the middle, and the extracted profiles are shown in the right. The height of the above the microstructures on the molds are 55µm, 55µm and 2µm, respectively. In addition, it is worth pointing out that the top of all the molds are quite smoothly (around Ra 10nm measured by Olympus Lext OLS4100).

2. Microstructure of Molds after GMP

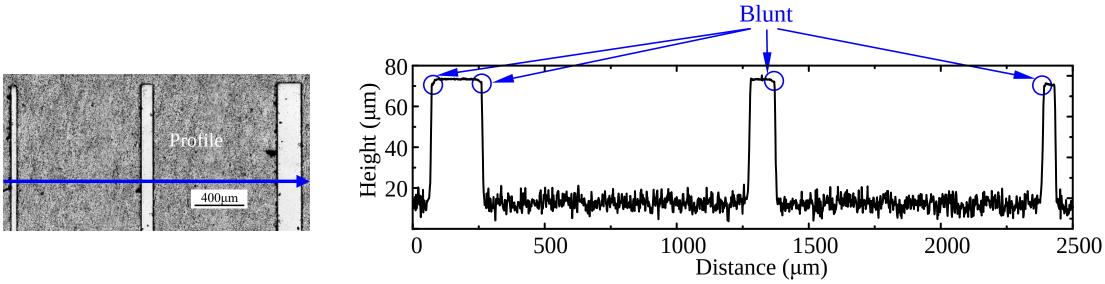

(a) Laser image (b) Profile perpendicular to the protrusions

Figure S3. The mold morphology after glass molding process (GMP). (**a**) Laser image; (**b**) Profile perpendicular to the protrusions.

In order to achieve mass production in the future, it necessitates to study tool wear during GMP. The mold morphology after GMP for 20 times is shown in Figure S3. The laser image is shown in Figure S3(a), while the corresponding profile is shown in Figure S3(b). Compared with the morphology before GMP, as shown in Figure S1, there is no obvious difference between them, and the shape and height are almost the same. It is worth pointing out that some small blunt are witnessed around the corner of the mold after GMP, as shown in Figure S3(b).

In order to investigate the microstructure of the blurs, the scanning electron microscope (SEM) measurement is conducted on the protrusion with the width of 200µm, 100µm and 50µm before GMP, and they are shown in Figure S4. It can be observed that the side wall of the protrusion is steep, and it is much rougher than its top surface due to its fabrication process. In addition, all the edge of the protrusion is quite sharp.

The microstructure images of molds after GMP are shown in Figure S5. The macro images with width of 200µm, 100µm and 50µm are demonstrated in Figure S5(a), Figure S5(b) and Figure S5(c), respectively. Compared with the morphology in Figure S4, it can be found that the macro shapes are almost the same, while the edges of the molds become blunt to some extent. The correspondingly enlarged images of the corners are shown in Figure S5(d), Figure S5(e) and Figure S5(f). The corner is supposed to experience severe stress and temperature during GMP alternately. When the pressing

step is conducting, the mold will press the glass with relatively large load at high temperature; while the load is reduced to its one sixth to maintain the molded shape when the annealing step is conducted; then, the load is taken off during the fast cooling step. The circle keeps on and on when GMP is conducting. The edges of the mold are sharp which reduces its stiffness, and makes them easier to deform during GMP. In terms of the areas away from the edges, it can maintain good shapes during GMP. Nevertheless, the small blunt of the edge will not influence the function performance of the molded glass. In addition, the results of energy-dispersive X-ray spectroscopy (EDS) analysis found that there was no adhesive wear on the surface of molds, which is another evidence that the mold was suitable for GMP.

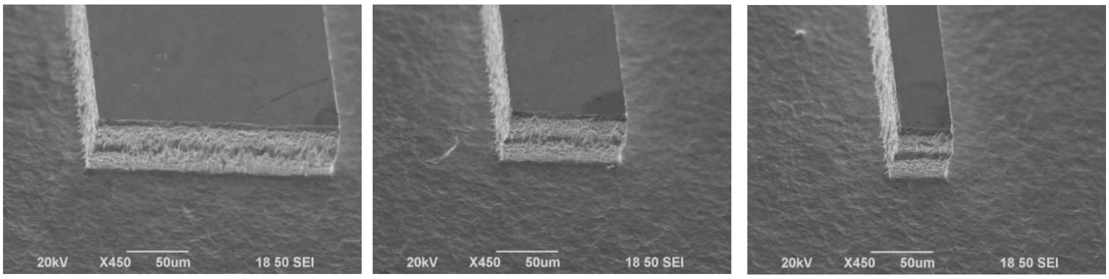

(a) 200μm width (b) 100μm width (c) 50μm width

Figure S4. The microstructure of molds before GMP. (**a**) 200μm width; (**b**) 100μm width; (**c**) 50μm width.

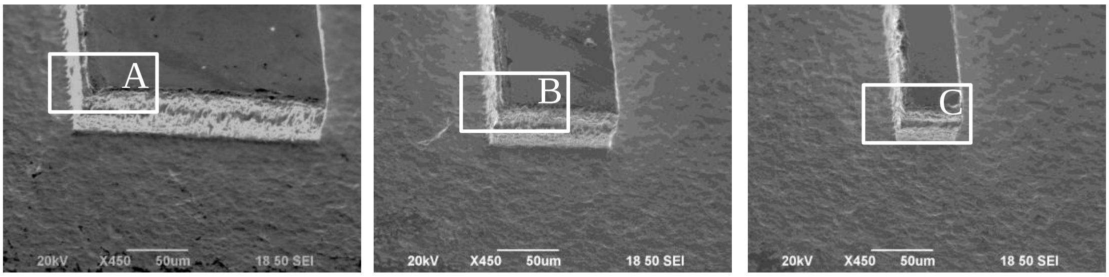

(a) 200μm width (b) 100μm width (c) 50μm width

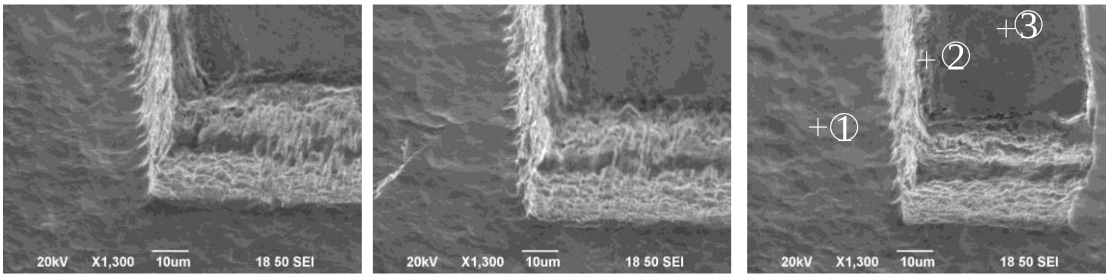

(d) Enlarged area A (e) Enlarged area B (f) Enlarged area C

Figure S5. The microstructure of the molds after GMP. (**a**) 200μm width; (**b**) 100μm width; (**c**) 50μm width; (**d**) Enlarged area A; (**e**) Enlarged area B; (**f**) Enlarged area C.
